# Evaluation of two family-based intervention programs for children affected by rare disease and their families – research network (CARE-FAM-NET): study protocol for a rater-blinded, randomized, controlled, multicenter trial in a 2x2 factorial design

**DOI:** 10.1186/s12875-020-01312-9

**Published:** 2020-11-20

**Authors:** Johannes Boettcher, Bonnie Filter, Jonas Denecke, Amra Hot, Anne Daubmann, Antonia Zapf, Karl Wegscheider, Jan Zeidler, J.-Matthias Graf von der Schulenburg, Monika Bullinger, Miriam Rassenhofer, Michael Schulte-Markwort, Silke Wiegand-Grefe

**Affiliations:** 1 Department of Child and Adolescent Psychiatry, Psychosomatics and Psychotherapy, University Medical Center Hamburg-Eppendorf, Hamburg, Germany; 2 Department of Pediatrics, University Medical Center Hamburg-Eppendorf, Hamburg, Germany; 3 Department of Medical Biometry and Epidemiology, University Medical Center Hamburg-Eppendorf, Hamburg, Germany; 4 Center for Health Economics Research Hannover (CHERH), Leibniz University Hannover, Hannover, Germany; 5 Institute for Medical Psychology, Center for Psychosocial Medicine, University Medical Center Hamburg-Eppendorf, Hamburg, Germany; 6 Department of Child- and Adolescent Psychiatry and Psychotherapy, Ulm University Hospital, Ulm, Germany

**Keywords:** Rare diseases, Family intervention, Internet-based intervention, Mental health, Randomized controlled trial

## Abstract

**Background:**

Families of children with rare diseases (i.e., not more than 5 out of 10,000 people are affected) are often highly burdened with fears, insecurities and concerns regarding the affected child and its siblings. Although families caring for children with rare diseases are known to be at risk for mental disorders, the evaluation of special programs under high methodological standards has not been conducted so far. Moreover, the implementation of interventions for this group into regular care has not yet been accomplished in Germany. The efficacy and cost-effectiveness of a family-based intervention will be assessed.

**Methods/design:**

The study is a 2x2 factorial randomized controlled multicenter trial conducted at 17 study centers throughout Germany. Participants are families with children and adolescents affected by a rare disease aged 0 to 21 years. Families in the face-to-face intervention CARE-FAM, online intervention WEP-CARE or the combination of both will be treated over a period of roughly 6 months. Topics discussed in the interventions include coping, family relations, and social support. Families in the control condition will receive treatment as usual. The primary efficacy outcome is parental mental health, measured by the Structured Clinical Interview for DSM-IV (SCID-I) by blinded external raters. Further outcomes will be assessed from the parents’ as well as the children’s perspective. Participants are investigated at baseline, 6, 12 and 18 months after randomization. In addition to the assessment of various psychosocial outcomes, a comprehensive health-economic evaluation will be performed.

**Discussion:**

This paper describes the implementation and evaluation of two family-based intervention programs for Children Affected by Rare Disease and their Family’s Network (CARE-FAM-NET) in German standard care. A methodologically challenging study design is used to reflect the complexity of the actual medical care situation. This trial could be an important contribution to the improvement of care for this highly burdened group.

**Trial registration:**

German Clinical Trials Register: DRKS00015859 (registered 18 December 2018) and ClinicalTrials.gov: NCT04339465 (registered 8 April 2020).

**Protocol Version:** 15 August 2020 (Version 6.1).

**Trial status:** Recruitment started on 1 January 2019 and will be completed on 31 March 2021.

**Supplementary Information:**

The online version contains supplementary material available at 10.1186/s12875-020-01312-9.

## Background

A disease is considered rare if no more than 5 out of 10,000 people are affected by it [1]. It is estimated that approximately 2 million children and adolescents in Germany live with one of up to 8000 different rare diseases [2]. While rare diseases comprise a heterogeneous group of complex disease patterns, they are usually characterized by a severe, chronic course, limited life expectancy and occurrence of symptoms already in early childhood [3]. Disease management requires a high degree of support and care from parents and siblings leading to high demands on the family [4]. Children and adolescents with rare diseases, their siblings and parents are therefore often under high physical and psychological stress [5]. Parents of chronically ill children are at high risk for clinical depression and anxiety [6, 7] and affected children themselves are at increased risk for externalizing and internalizing behavioral, as well as developmental disorders [8]. Siblings grow up in a living environment that is characterized by the care and medical treatment of their sick sibling. They are often involved in the responsibility for their siblings, in nursing, and technical medical tasks. Therefore, it is not surprising that siblings also exhibit an increased risk for behavioral disorders [9, 10]. Despite their high level of stress, all family members must “function” to a high degree and organize care for the sick child. Even under great stress and with psychological symptoms, parents usually do not seek conventional psychosocial care, e.g. psychotherapy for their children or themselves, because this would require additional time and emotional resources [11]. Although psychosocial care in the form of conciliar consultation is available in German children’s hospitals, this cannot offer effective, comprehensive treatment.

In view of the foregoing, it is evident that there is a high need for studies with validated outcome measures, high methodological quality, rigorous evaluation designs, high quality cost data and sufficient sample size to implement feasible and acceptable interventions for this at risk group [12]. To address this demand, a family-based psychodynamic intervention program Children Affected by Rare Disease and their Families (CARE-FAM) and a Web-Based Psychological Support Program for Caregivers (WEP-CARE) will be implemented and evaluated in a randomized, controlled multicenter trial. CARE-FAM is a low-frequency family-oriented intervention that is based on the development model of Mattejat and colleagues (2000) [13] and the family counselling approach for children with mentally ill parents [14]. WEP-CARE, in contrast, is a web-based therapy for severely distressed parents of children with chronic diseases, based on cognitive-behavioral writing therapy [15].

### Study objectives

The central objectives of CARE-FAM-NET are the implementation, accompanying evaluation and in case of success transfer into standard care of the two novel psychosocial types of care CARE-FAM and WEP-CARE for children with rare diseases, their siblings and parents at 17 locations in Germany. The focus is on a psychosocial intervention that is individually and precisely tailored to the respective family and meets the following requirements: cross-sectoral, needs- and family-oriented, interdisciplinary and multidisciplinary. Existing care structures in children’s hospitals and centers for rare diseases are used, connected with CARE-FAM-NET and extended by qualified psychosocial care. Moreover, an important element of this study design is the comprehensive evaluation of the intervention’s cost-effectiveness. The objective is to improve the mental health and quality of life of all family members: parents as well as affected children and siblings.

## Methods/design

The Standardized Protocol Items Recommendations for Interventional Trials (SPIRIT) was used [16] to ensure a structured description of the trial presented and that all relevant information are included. A corresponding SPIRIT checklist is given in Supplementary Table 1. Moreover, all items from the World Health Organization Trial Registration Data Set are shown in Supplementary Table 2.

### Study design

The study is a prospective, 2x2 factorial randomized controlled multicenter superiority trial. With assessments at baseline as well as at 6, 12, and 18 months after randomization, the family-based intervention programs CARE-FAM, WEP-CARE and the combination CARE-FAM + WEP-CARE are evaluated against a control group in terms of their effectiveness and efficacy. All families have the possibility to use routine care services. Therefore, families in the control group receive treatment as usual (TAU), meaning they do not receive any intervention within this study. Outcomes will be assessed from the parents’, patient’s, and siblings’ perspective as well as from the therapist’s perspective. The primary outcome of the study will be assessed by external, blinded raters.

### Study setting

The following 17 centers, which are located all over Germany, are involved in the evaluation of the program: Augsburg (Augsburg Hospital and Josefinum KJF Clinic), Berlin (University Hospital Charité Berlin), Berlin (Academic Hospital DRK Berlin Westend), Bielefeld (Evangelical Hospital Bethel Bielefeld), Bochum (University Medical Center Ruhr-Bochum), Cologne (University Hospital Cologne), Essen (Essen University Hospital and LVR Clinics Essen), Freiburg (Medical Center – University of Freiburg), Gießen (University Hospital of Gießen), Göttingen (University Medical Center Göttingen), Hamburg (University Medical Center Hamburg-Eppendorf), Hannover (Hannover Medical School), Homburg (Saarland University Hospital), Jena (Jena University Hospital), Leipzig (University of Leipzig Medical Center), Münster (Münster University Hospital), Rostock (Rostock University Medical Center). All participating study centers will be involved in patient recruitment, diagnostics and in the implementation of the intervention. As a partner Ulm (Ulm University) will be responsible for the coordination and implementation of the online intervention. The health economic evaluation will be carried out by the Center for Health Economics Research Hannover (CHERH, Leibniz University Hannover) and the quality management by the aQua-Institute. The study center in Hamburg will additionally be responsible for the coordination of the study. An independent contract research organization (CTC North, Hamburg, Germany) will conduct the data management and data monitoring. A steering committee at the coordinating center in Hamburg (SWG, JB, BF, JD, AZ) as well as an external scientific advisory board will regularly examine the study’s progress. Yearly meetings of the scientific advisory board and monthly meetings of the steering committee will take place. In these meetings, the study progress, the recruitment, any protocol deviations, the loss to follow-up, serious adverse events and any problems of the study conduct will be discussed.

### Participants

Participants will be recruited from in- and outpatient departments of pediatric hospitals in the 17 participating study centers. Pediatricians will be asked to inform their rare disease patients and the patients’ families about the project and encourage them to contact the study team. Once families have expressed their interest to participate in the study, a member of the research staff will approach the respective families and inform them about the project.

Once all participating family members have provided written informed consent, the baseline questionnaires and interviews will be conducted. An overview of the inclusion and exclusion criteria is given in Table 1. Families with at least one child with a rare disease aged from 0 to 21 years will be included. Families can decide for themselves which family members want to participate; however, all family members will be encouraged to take part in the study. Both couples as well as single parents can take part in the study. Participation is not limited to biological parents; adoptive parents, stepparents as well as foster parents may enter in the study. To compensate for their time conducting the interviews and filling in the questionnaires, each family will receive a financial compensation of €50 if all follow-up assessments are completed.

**Table 1:** Inclusion and exclusion criteria

**Inclusion criteria**	
1. Family with at least one child between the age of 0 and 21 years with a rare disease or suspected rare disease 2. Written informed consent with the study protocol 3. Sufficient knowledge of the German language of parents and children 4. Insured at the participating insurance companies	
**Exclusion criteria**	
1. Severe psychiatric disorders and impairments with acute symptoms such as suicidal tendencies, massive self-harming behavior, acute psychotic symptoms etc., which will not be adequately addressed by these novel low-frequency interventions	

### Interventions

#### Care-FAM

The face-to-face intervention CARE-FAM is a manualized family-based intervention for the diagnosis, early detection and early treatment of mental health issues of children affected by rare diseases, their siblings and their parents. CARE-FAM-NET is based on the predecessor project CHROKODIL, a psychodynamic intervention for families with a chronically ill children [17]. It is a brief low-frequency intervention comprising six to eight semi-structured sessions (60 min) per family over a period of six months. Upon request, the sessions can take place at the family’s home (home-treatment). The program begins with a preliminary talk about the procedure and topics of the CARE-FAM program. The following two to three sessions with the parents include communication and information about the disorder as well as the coping with the disorder within the family. At the same time, the couple’s relationship, the children and the parent-child-relationship are examined; the parent’s families of origin, contact with other family caregivers and family living conditions are also looked at. This information serves as a starting point for the family-based intervention. Children and adolescents are subsequently seen for one to two individual sessions per child. The main objective is to capture the family situation, focusing on individual and familial coping as well as relationship structures inside and outside the family.

The program is concluded with about three sessions for the whole family, which are the core of the CARE-FAM intervention. These sessions include all family members and aim to bring together all individual perspective’s findings from the single sessions. At the same time, openness, transparency and communication within the whole family can be encouraged. The family’s present and future handling of the disease and support from inside and outside the family can be reflected on and responded to. Depending on the family’s needs and wishes, individual topics like current conflicts can also be discussed. If any family member or the whole family needs further treatment or support, additional aids will be initiated by the program staff. If participants agree, all intervention sessions will be video-recorded for quality control reasons. All therapists are experienced in the field of adult or child and adolescent psychiatry and received a comprehensive two-day training based on the CARE-FAM manual. Adherence to the intervention manual will be enhanced by continuous supervision. During the corona pandemic, in-person therapy sessions will be replaced by online video sessions whenever possible.

The goals of the CARE-FAM program can be structured into the three subsequent levels: coping, relationships and family dynamics. On the (1) coping level, the main aims are the reinforcement of coping strategies for the handling of the child’s disease and discussions about important events maintaining conflicts inside the family, such as hospitalization, loss of employment or change of residence. On the (2) relationship level, the main aims are to overcome social isolation and strengthen intra-familial relationships. On the level of (3) family dynamics, the main aims are to promote the understanding of the disease and the couple and family dynamics from a psychodynamic, multi-generational perspective.

#### WEP-care

WEP-CARE is a manualized supportive online intervention addressed to parents of chronically ill children and adolescents [15, 18, 19]. WEP-CARE is based on well proven principles of cognitive-behavioral writing therapy and is conducted entirely via the internet. The aim of the intervention is to support parents with the psychosocial management of their child’s disease. By reducing parents’ psychological complaints such as anxiety symptoms, depressive symptoms or stress symptoms, quality of life as well as the whole family’s ability to cope with the disease will be improved. In the long term, parents should master everyday life with a chronically ill child more easily. As the intervention reduces the psychological burden of the parents, it will also have positive effects on the affected children and their siblings.

With the support of therapists trained in this program, the participants carry out 12 standardized writing tasks of about 45 min at approximately weekly intervals in a time period of 12 to 14 weeks, on a data-secured internet platform. The therapists give individualized feedback within 48 h with further instructions. This feedback is supervised within the framework of continuous supervision to guarantee adherence to the intervention manual. The main focus of the intervention lies on overcoming fear. Within the writing tasks well established behavioral and cognitive techniques are used, as for example exposition, cognitive restructuring or problem-solving training. Furthermore, by establishing positive activities, resource activation and drawing attention on positive occurrences concerning the disease, depressive symptoms are also addressed. The following topics are covered within the 12 writing tasks of the program: current handling of the disease, overcoming fear, problem solving, self-care and stabilization as well as integration of what has been achieved. WEP-CARE was successfully evaluated in a pilot study with parents of children affected by cystic fibrosis [15, 20] and implemented in a generic adaptation for parents of chronically ill children. Results showed large effects of anxiety reduction and medium effects of improved depression and quality of life of affected parents.

#### Control group

Families who have been randomly assigned to the control condition will receive TAU. They will receive the standard treatment that is normally given in regular care but no additional intervention within this study. All participating families in the control group and intervention groups are not prohibited from seeking additional treatment or additional interventions outside of the study.

### Outcome measures

Sociodemographic information including age, gender, number of children, educational and employment status, somatic and psychiatric diseases, history of treatment and current treatment will be recorded with a specifically designed questionnaire at baseline.

#### Primary outcomes

Parent’s psychopathology at 18 months after randomization will be measured by the German version of the Structured Clinical Interview for DSM-IV Axis I Disorders (SCID-I) [21]. The SCID-I is a standardized semi-structured interview for diagnosis of major mental disorders according to DSM-IV Axis I. A trained external rater (blind to the family’s group assignment) will perform the SCID-I by interviewing the parents on their psychopathology.

#### Secondary outcomes

The parents’ health-related quality of life will be assessed by the German version of the EuroQoL-5 (EQ-5D) [22–24]. The quality of life in parents will be assessed by the Ulm Quality of Life Inventory for Parents (ULQIE) [25]. In addition, quality of life in parents will be assessed with the 12-Item Short Form Health Survey (SF-12) [26]. The health-related quality of life of the diseased children will be assessed by the DISABKIDS Chronic Generic Measure-37 (DCGM-37) [27]. The health-related quality of life of both diseased children and their siblings will be assessed by the KIDSCREEN-27 [28]. A short version of the KIDSCREEN-27, the KIDSCREEN-10, will be used for the calculation of quality-adjusted life years for children and adolescents [29, 30].

The parent’s symptomatology will be assessed by the German version of the Brief Symptom Inventory (BSI) [31], a short version of the Symptom Checklist-90-Revised (SCL-90-R). In addition, the German version of the Patient Health Questionnaire (PHQ-D) [32] will be applied to screen for depressive, anxiety, somatoform, alcohol, and eating disorders. Further aspects of the parental psychopathology will be assessed by the German version of the Global Assessment of Functioning (GAF) [33], which is used to rate a person’s overall level of functioning.

The children’s psychopathology will be assessed using the German version of the Kiddie Schedule for Affective Disorders and Schizophrenia Present and Lifetime Version (K-SADS-PL) [34]. The K-SADS-PL is a standardized semi-structured interview for an early diagnosis of mental disorders in children and adolescents according to ICD-10 and DSM-IV criteria. Interviews on the children’s psychopathology will be conducted about each child using the parent-proxy version as well as the self-report version for children 10 years or older. In addition, the children’s global level of functioning will be assessed by the German version of the Children Global Assessment Scale (CGAS) [35].

Children’s psychopathology will further be assessed by the German version of the Child Behavior Checklist (CBCL) [36] and the Youth Self Report (YSR) [37]. Both questionnaires describe a variety of internalizing and externalizing behaviors in children.

Coping of the parents will be assessed with the Coping Health Inventory for Parents [38] and coping strategies of the children will be assessed with the KidCOPE [39].

The Oslo Social Support Scale (OSSS) [40] will be used to measure social support of both parents and children. Family functioning and relational health of the family will be rated on the German version of the Global Assessment of Relational Functioning Scale (GARF) [33].

The quality of sibling relationships will be assessed by the siblings of the rare diseased child using the German version of the Sibling Relationship Questionnaire (SRQ) [41] in the self-report version. The quality of the parental relationship will be assessed using the self-reported Relationship Satisfaction Questionnaire (“Partnerschaftsfragebogen”, PFB) [42].

Body-related disorders in the rare diseased child will be assessed using the German versions of the Eating Disorders in Youth-Questionnaire (EDY-Q) [43], the 8-item short form of the Eating Disorder Examination Questionnaire for Children (ChEDE-Q8) [44], and the Questionnaire on Enuresis and Functional Urinary Incontinence (“Anamnesebogen zu Enuresis und funktioneller Harninkontinenz”) [45].

The assessment of psychosocial and health care service use as a basis for the estimation of treatment costs for the parents will be performed by the German version of the Client Socioeconomic and Services Receipt Inventory (CSSRI-DE) [46, 47]. The CSSRI-DE was developed for cost analysis of the psychiatric supply system und allows a comprehensive assessment of all substantial components for direct and indirect costs. The German version of the Children and Adolescent Mental Health Services Receipt Inventory (CAMHSRI) [46] will be used to assess the health and psychosocial service use as a basis for the assessment of treatment costs for the children.

Objectives of the treatment and the achievement of these objectives will be assessed by ad hoc items designed by the study group. The evaluation of the treatment will be assessed by the Therapy Evaluation Questionnaire (“Fragebogen zur Beurteilung der Behandlung”, FBB) [48], which assesses the subjective quality of care according to the two main aspects quality of results (treatment success) and process quality (treatment procedure). Satisfaction of the families with their participation in the study will be assessed using the Client Satisfaction Questionnaire (“Fragebogen zur Messung der Patientenzufriedenheit”, ZUF-8) [49]. The systematic examination of the psychotherapy process will be assessed with the German Version of the Vanderbilt Psychotherapy Process Scales (VPPS) [50].

All interviews and questionnaires will be administered at baseline as well as at 6, 12 or 18 months after randomization. A trained blinded external rater will perform the interviews, which will be audiotaped for quality control. Raters will remain blinded regarding the family’s group assignment over the course of the study. All other questionnaires will be filled out by the participants themselves. The measures to be administered at each time point are listed as SPIRIT schedule in Table 2.

**Table 2:** Outcomes and measures

Outcome		Instrument		Source						Time (months)			
		Questionnaire	Interview	Mother	Father	Diseased child	Sibling	Therapist	External rater	pre	6	12	18
Demographics		Ad-hoc Items		X	X					X			
Quality of Life	Parents	EQ-5D		X	X					X	X	X	X
		ULQIE		X	X					X	X	X	X
		SF-12		X	X					X	X	X	X
	Diseased Child	DCGM-37		X	X	X				X	X	X	X
	Sibling	Kidscreen-27		X	X	X	X			X	X	X	X
Diagnoses/Psychiatric symptomatology	Parents		SCID-I	X	X				X	X	X	X	X
		PHQ-D		X	X					X	X	X	X
		BSI		X	X					X	X	X	X
		GAF							X	X	X	X	X
	Children		K-SADS-PL						X	X	X	X	X
		CGAS							X	X	X	X	X
		CBCL 1½–5 / CBCL 6-18R / YSR		X	X	X	X			X	X	X	X
Coping	Parents	CHIP-D		X	X					X	X	X	X
	Children	KidCOPE				X	X			X	X	X	X
Social network	Social support	OSSS		X	X	X	X			X	X	X	X
	Family relations	GARF							X	X	X	X	X
	Sibling relationship	SRQ					X			X	X	X	X
Partnership satisfaction	Parent relationship	PFB		X	X					X	X	X	X
Body-related disorders (Diseased child)	Eating Behavior	EDY-Q		X	X	X				X	X	X	X
	Body related eating behavior	ChEDE-Q8				X				X	X	X	X
	Excretion Disorders	Questionnaire on Enuresis and Functional Urinary Incontinence		X	X					X	X	X	X
Health economics	Intervention costs children		CAMHSRI-DE	X	X				X	X	X	X	X
	Intervention costs parents		CSSRI-DE	X	X				X	X	X	X	X
Diagnostics of care and quality of care	Previous experience and treatment expectations	Ad-hoc Items		X	X	X	X			X	X	X	X
	Treatment goals	Ad-hoc Items		X	X	X	X	X		X	X	X	X
	Satisfaction- and treatment assessment^a^	FBB		X	X	X	X	X			X	X	X
		ZUF-8		X	X	X	X				X	X	X
		VPPS^a^									X		
		Ad-hoc Items		X	X	X	X	X			X	X	X

^a^intervention groups only (CARE-FAM, WEP-CARE)

#### Sample size

Sample size calculation was carried out with the software PASS 2015, using two independent proportions and is based on the assumed increase in the proportion of non-conspicuous parent among the initially conspicuous parents according to SCID-I criteria between the patients treated with CARE-FAM and patients that did not receive CARE-FAM at18 months after randomization. Additionally, we assume the same effect size between patients with and without WEP-CARE. Furthermore, we suppose that the two interventions work independently of each other. Given a power of 80% and a type I error rate of 5% (two-sided hypothesis), 74 initially conspicuous parents per treatment combination group (296 in total) are needed to detect a difference of 11.2% (91.2% of the CARE-FAM patients and 80% of the non-CARE-FAM patients) in the proportion of non-conspicuous parents between both groups. We further assume that at the beginning of the study 30% of the parents are conspicuous. So, at the end of the study, a total of 988 parents must be available for evaluation. Additionally, we expect a dropout rate of 20% between baseline and follow up. To compensate for this, a total of 1236 parents must be recruited. This means that a total of 618 (rounded up to 620) families (310 per stratum CARE-FAM yes/no, 155 families per group of the four randomization groups: CARE-FAM only, WEP-CARE only, combination, no study intervention) has to be included in the trial.

### Research procedure

In order to be able to realistically recruit the sample, recruitment will be extended to several clinics and outpatient clinics. Patients will be recruited at the above-mentioned cooperating clinics and outpatient clinics, where approved therapists can treat study patients. The project staff members will review the weekly admissions and discharge lists and/or participate in the outpatient clinic/ward conferences of the cooperating institutions. The project staff will inform eligible patients and their parents about the study and ask them to participate in the study. If informed consent is given, diagnostics will be carried out.

#### Randomization and blinding

Computerized lists for familywise randomization with variable block length stratified by recruitment centers are prepared by the Department of Medical Biometry and Epidemiology of the University Medical Center Hamburg-Eppendorf. According to a 2x2 factorial study design, each family is randomized to one of four combinations of the two interventions (CARE-FAM only, WEP-CARE only, combination, no study intervention). The subsequent central allocation procedure will be managed by the coordinating study center in Hamburg to guarantee allocation concealment. Randomization will take place once the standardized baseline diagnostics are complete. Thus, every randomized family received comprehensive standardized diagnostics of every participating child and parent. This will maintain the families’ interest in the study and enhance retention. Although families will be aware of the group they have been assigned to, the external raters assessing the primary outcome and the health-economics will be blinded regarding the families’ group allocation to avoid detection bias by project members.

#### Data assessment and data management

**Fig. 1:**
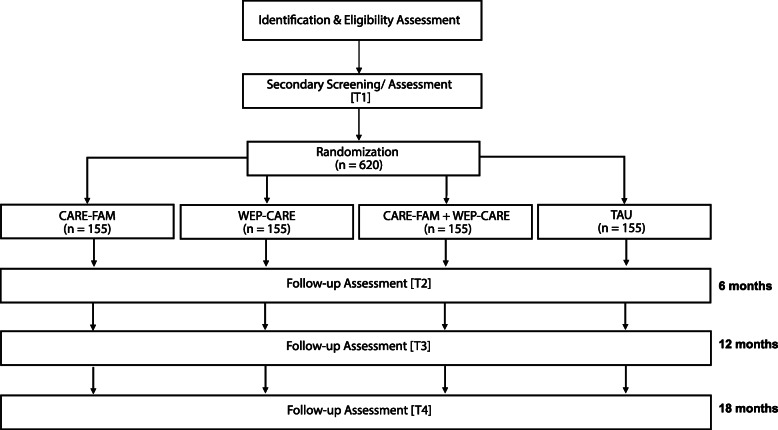
Study design

The study design is illustrated in Fig. 1. At the beginning of the study, after signing the informed consent forms, the parents caring for a child with rare diseases will fill in the baseline questionnaires and participate in the structured clinical interviews, SCID-I about themselves and K-SADS-PL about their child/children. All children aged 10 years and older will also be asked to complete this diagnostic process if possible. As soon as all documents are complete, the family will be randomized into one of four groups. The research staff will subsequently inform the family about their group assignment and provide feedback on the results of the diagnostic interviews.

If the family is assigned to the intervention group, the therapist who carries out the CARE-FAM and/or WEP-CARE program will be informed, and the start of the intervention will be initiated. If the family is assigned to the control group, no further treatment will be provided within the CARE-FAM study.

At 6 (T2), 12 (T3) and 18 (T4) months after randomization, the families will be contacted again by the research staff. The majority of questionnaires and diagnostic interviews for all three follow-up assessments are the same as at the baseline assessment. Furthermore, after 18 (T4) months external medical data (e.g. treatment data) and cost data from the participating health insurance companies is requested.

For quality assurance, site visits will be conducted in all study centers by principal investigators of CTC North to monitor study progress and compliance with the predetermined regulations of the study.

Study data of each study center will be collected and managed by the study center in Hamburg. The study center Hamburg will receive the original and scanned files from all other study centers. The electronic file will remain in the respective study center. Data will be collected on paper and entered into the electronic case report form (eCRF) provided by secuTrial®, which is designed by CTC North. This data entry system will be password-protected and accessible only to the database managers and study team. To ensure complete and accurate data, visual inspection of the data collected on paper will be carried out before data entry. Trained personnel will carry out the data collection via double entry. Afterwards a third person will review the double data entry. In cooperation with CTC North automated rules for detecting inconsistencies in the data will be integrated and validated by a defined Data Validation Plan.

All study-related information will be stored securely at the respective study center. All records that contain names or other personal identifiers, such as informed consent forms, will be stored separately from study records identified by code numbers. The coordinating study center will oversee the intra-study data sharing process.

All study outcomes, will be published in peer-reviewed journals and presented at scientific conferences. Authorship will be based on the eligibility criteria defined by the International Committee of Medical Journal Editors. All papers and abstracts must be approved by the coordinating center in Hamburg before submission.

Any change to the protocol which may impact the conduct of the study (e.g. study design, patient population, sample sizes, study procedures) will require a formal amendment. Any such amendment will be agreed upon by Deutsches Zentrum für Luft- und Raumfahrt (DLR), and approved by the local Ethics Committee (Medical Chamber Hamburg) prior to implementation.

In this study, serious adverse events are defined as (a) death or suicide, (b) self-harm, (c) hospitalization or prolongation of existing hospitalization, or (d) distress or attendance at emergency department, whereas adverse events are defined as any deterioration of the mental state and any perception of the patient to have been adversely affected. Serious adverse events and adverse events will be compiled after the families have given consent and enrolled in the study and will be recorded by the study personnel.

### Data analysis

For both, primary and secondary outcomes, descriptive statistics are presented separated by treatment groups on family level as well as on child and parents’ level. Categorical variables are summarized by absolute and relative frequencies. Continuous variables are summarized by mean and standard deviation (SD) or by median, quartiles and/or interquartile range (IQR), as appropriate. The number of available observations and the number of missing observations are reported separately for treatment groups.

The primary outcome, parent’s psychopathology measured by the SCID-I, is evaluated according to the intention-treat (ITT) principle. The ITT population consists of all randomized families for which baseline documentation is available. As the members of a family live under common conditions and interact with each other, cluster effects can be assumed. Thus, the inferential statistics are performed with mixed models. The primary outcome is binary (diagnosis according to SCID-I yes/no). A mixed logistic regression with family and family member as random effects, recruitment center, therapy group (2 factors: 1st factor CARE-FAM yes/no, 2nd factor WEP-CARE yes/no), and time as fixed effects within the ITT population of all initially conspicuous parents is performed. The interactions between time and the therapy groups is determined. If they are not significant, they are eliminated from the model. Missing values are treated by direct imputation to allow an ITT analysis. Under the assumption of two stochastically independent interventions, the contrast of each group factor at T4 is tested at the 5% level (two-sided hypothesis). Thereby, each intervention can prove to be effective on its own. Only these results are interpreted in a confirmatory manner. In an additional analysis, we test the assumption of independence by examining the interaction between both therapy group factors. The primary analysis is repeated in the per protocol population, which consists of all families with at least three sessions in CARE-FAM or WEP-CARE. To assess the effect of the interventions within specific subgroups, the following subgroup analyses are performed regarding study center, disease of children, age of the children, and disease of parents. In a series of sensitivity analyses, missing values are imputed by different approaches as last observation carried forward, multiple imputation, worst- and best-case imputations. Secondary outcomes are investigated exploratively without adjustment for multiplicity in the ITT population. The analysis strategy for the secondary, binary outcomes is the same as in the primary analysis. For secondary, continuous outcomes, linear mixed models with differences to baseline as outcome, family and family member as random effects and the same fixed effects as in the above-mentioned model and respective baseline value as covariate are examined. The safety outcomes are determined using frequency tables and if possible, with mixed logistic regression analogue to the primary analysis. Odds ratios or adjusted means, their 95% confidence interval, and *p* values are reported according to the CONSORT statement. Interim analyses are not planned. Statistical analyses will be carried out with standard statistical software.

## Discussion

This study presents an opportunity to implement two family-based intervention programs for families caring for children with rare diseases in German standard care. In the current trial, the program’s effectiveness and cost-effectiveness will be evaluated in a randomized-controlled design with a comprehensive health-economic assessment at baseline, 6, 12, and 18 months.

An important strength of this study is the use of a randomized controlled trial design with a combination of both interventions (CARE-FAM + WEP-CARE) and a control group receiving TAU, meaning no intervention within this study. This will be a stronger test of the intervention’s effectiveness. Moreover, the study design will include a comprehensive health-economic evaluation. The broad nature of the inclusion criteria will enable families of patients with a variety of rare diseases to take part in the study.

A wide range of psychosocial outcomes for both children and parents (e.g., mental health, health-related quality of life, coping, social support, family relationships) will be assessed. The outcomes concerning the children will not only be measured from the parents’ perspective; self-ratings of the children will also be obtained for all children aged 10 years and older. Most importantly, the parents’ and children’s mental health will be assessed by an external rater blind to the family’s assigned group.

This extensive assessment of outcomes will allow for a comprehensive analysis of various factors influencing the interplay of the mental health between the parents and their children.

An important implication for practice is that all family members participating in the study will be screened for mental health problems at the very beginning of study participation. This will provide an opportunity for early detection of mental illness, for which appropriate offers of assistance can subsequently be recommended.

Difficulties could arise from the multicenter design of this study. The combination of both university and supply clinics could lead to differences in the recruitment process. Hence, possible center effects will be statistically taken into account.

Moreover, the standardised execution of the manualised face-to-face intervention might prove difficult. A great number of therapists from different therapeutic approaches will conduct the CARE-FAM program. To ensure the implementation according to the manuals, the process and the methods of each sessions and the writing tasks will be documented and later analysed for compliance with the manuals.

Experience from this trial will be used to revise the existing manual. The financing of the intervention in regular care beyond the term of the study will be discussed with health-insurance representatives during the term. As a result, we aim to not only evaluate the CARE-FAM and WEP-CARE intervention in the participating study centers, but to also implement it into regular care throughout Germany.

The support provided to families caring for children with rare diseases should be comprehensive and integrative and therefore be oriented towards all family members, as the psychosocial and medical care for the affected families is equally complex. If successful, both interventions could be the first programs for affected families to be implemented into regular practice nationwide in Germany; this could be an important, future-oriented contribution to the improvement of a family-oriented support system for this particularly burdened group.

## Supplementary Information

**Additional file 1.**

**Additional file 2.**

## Data Availability

The datasets generated during the current study will be made available in the repository of ClinicalTrials.gov (NCT04339465) once the data entry is complete.

## Abbreviations

BSI: Brief Symptom Inventory
CAMHSRI: Children and adolescent mental health services receipt inventory
CARE-FAM: Children Affected by Rare Disease and their Families
CBCL: Child Behaviour Checklist
ChEDE-Q8: Eating Disorder Examination Questionnaire for Children
CSSRI-DE: Client Socioeconomic and Services Receipt Inventory
DCGM-37: DISABKIDS Chronic Generic Measure-37
DSM-IV: Diagnostic and Statistical Manual for Mental Disorders, 4th Edition
EDY-Q: Eating Disorders in Youth-Questionnaire
EQ-5D: EuroQoL-5
FB-A: Allgemeiner Familienfragebogen
FBB: Fragebogen zur Beurteilung der Behandlung
FKV: Freiburger Fragebogen zur Krankheitsbewältigung
GAF: Global Assessment of Functioning
GARF: Global Assessment of Relational Functioning Scale
K-SADS-PL: Schedule for Affective Disorders and Schizophrenia for School Aged Children
OSSS: Oslo Social Support Scale
PHQ-D: Patient Health Questionnaire
SCID-I: Structured Clinical Interview for DSM-IV
SF-12: 12-Item Short Form Health Survey
SRQ: Sibling Relationship Questionnaire
TAU: Treatment as usual
ULQIE: Ulm Quality of Life Inventory for Parents
VPPS: Vanderbilt Psychotherapy Process Scales
WEP-CARE: Web-Based Psychological Support Program for Caregivers
YSR: Youth Self Report
ZUF-8: Fragebogen zur Messung der Patientenzufriedenheit
