# Supplementary material for: Evaluation of two family-based intervention programs for children affected by rare disease and their families – research network (CARE-FAM-NET): study protocol for a rater-blinded, randomized, controlled, multicenter trial in a 2x2 factorial design
Source: BMC Fam Pract. 2020 Nov 20;21:239. doi: 10.1186/s12875-020-01312-9 (PMC7678588; doi:10.1186/s12875-020-01312-9)
Supplement: Supplementary file 2 — Additional file 2. [file 12875_2020_1312_MOESM2_ESM.docx]

|  | **Data category** | **Information** |
| --- | --- | --- |
|  | Primary registry and trial identifying number | German Clinical Trials Register  DRKS00015859 |
|  | Date of registration in primary registry | 8 April, 2020 |
|  | Secondary identifying numbers | ClinicalTrials.gov NCT04339465 |
|  | Source(s) of monetary or material support | German Federal Joint Committee (G-BA) |
|  | Primary sponsor | Silke Wiegand-Grefe, Prof. Dr  University Medical Center Hamburg-Eppendorf, Germany |
|  | Secondary sponsor(s) | German Federal Joint Committee (G-BA) |
|  | Contact for public queries | Silke Wiegand-Grefe, Prof. Dr  University Medical Center Hamburg-Eppendorf, Germany  Martinistraße 52  KJP-Forschung, Building W35  20246 Hamburg  +49 407410 53603  silke.wiegand-grefe@uke.de |
|  | Contact for scientific queries | Silke Wiegand-Grefe, Prof. Dr.  University Medical Center Hamburg-Eppendorf, Germany  Martinistraße 52  KJP-Forschung, Building W35  20246 Hamburg  +49 407410 53603  silke.wiegand-grefe@uke.de |
|  | Public title | Children Affected by Rare Disease and Their Families Network (CARE-FAMNET) |
|  | Scientific title | Family-based intervention program for children affected by rare disease and their family’s network (CARE-FAM-NET): a randomized, controlled, multicenter trial |
|  | Countries of recruitment | Germany |
|  | Health condition(s) or problem(s) studied | Rare diseases (prevalence <5:10,000) |
|  | Intervention(s) | CAREFAM: face to face low-frequent short family intervention  WEP-CARE: parental online writing intervention  CARE-FAM + WEP-CARE: both interventions  TAU: treatment as usual |
|  | Key inclusion and exclusion criteria | *Inclusion Criteria*:  1. Family with at least one child between 0 and 21 years with a rare disease or a suspected rare disease  2. Consent to participate in the study  3. Sufficient knowledge of the German language of parents and children  4. Insured at the participating insurance companies  *Exclusion Criteria*: Severe psychiatric disorders and impairments with acute symptoms such as suicidal tendencies, severe depression, addictions, acute psychotic symptoms etc., which will not be sufficiently treated by this new low-frequency intervention. Children and parents with acute treatment demand in the control group will be placed at psychotherapists. Nevertheless, they stay in the control group. |
|  | Study type | Interventional Allocation: randomized Intervention model: factorial assignment Masking: blind - assessors are blind regarding the randomization (group affiliation) of the families Primary purpose: prevention |
|  | Date of first enrolment | January 2019 |
|  | Sample size | Target sample size: 620 families  Enrollment so far: 402 (status as of 23. Juli 2020) |
|  | Recruitment status | Recruiting |
|  | Primary outcome(s) | Mental health of parents (SCID)  (Time Frame: Change from baseline of the study at 6,12 and 18 months after the randomization; not designated as safety issue) |
|  | Key secondary outcomes | Health-related quality of life of the parents (EQ-5D)  (time frame: Change from baseline of the study at 6,12 and 18 months after the randomization; not designated as safety issue).  Health-related quality of life of the parents (ULQIE)  (time frame: Change from baseline of the study at 6,12 and 18 months after the randomization; not designated as safety issue).  Health-related quality of life of the parents (SF-12)  (time frame: Change from baseline of the study at 6,12 and 18 months after the randomization; not designated as safety issue).  Health-related quality of life of the parents (SF-12)  (time frame: Change from baseline of the study at 6,12 and 18 months after the randomization; not designated as safety issue).  Health-related quality of life of the chronically-ill children/adolescents (Kidscreen-27)  (time frame: Change from baseline of the study at 6,12 and 18 months after the randomization; not designated as safety issue).  Health-related quality of life of the chronically-ill children/adolescents (DCGM-37)  (time frame: Change from baseline of the study at 6,12 and 18 months after the randomization; not designated as safety issue).  Mental health of the parents (PHQ)  (time frame: Change from baseline of the study at 6,12 and 18 months after the randomization; not designated as safety issue).  Mental health of the parents (BSI)  (time frame: Change from baseline of the study at 6,12 and 18 months after the randomization; not designated as safety issue).  Mental health of the parents (GAF)  (time frame: Change from baseline of the study at 6,12 and 18 months after the randomization; not designated as safety issue).  Mental health of the chronically-ill children/adolescents and siblings (Kiddie-SADS-PL)  (time frame: Change from baseline of the study at 6,12 and 18 months after the randomization; not designated as safety issue).  Mental health of the chronically-ill children/adolescents and siblings (CBCL)  (time frame: Change from baseline of the study at 6,12 and 18 months after the randomization; not designated as safety issue).  Mental health of the chronically-ill children/adolescents and the siblings (YSR)  (time frame: Change from baseline of the study at 6,12 and 18 months after the randomization; not designated as safety issue).  Coping of the parents (CHIP-D)  time frame: Change from baseline of the study at 6,12 and 18 months after the randomization; not designated as safety issue).  Coping of the chronically-ill children/adolescents and the siblings (Kidcope)  (time frame: Change from baseline of the study at 6,12 and 18 months after the randomization; not designated as safety issue).  Social support of the parents, of the chronically-ill children/adolescents and of the siblings (OSSQ)  (time frame: Change from baseline of the study at 6,12 and 18 months after the randomization; not designated as safety issue).  Family functioning (GARF)  (time frame: Change from baseline of the study at 6,12 and 18 months after the randomization; not designated as safety issue).  Relationships between siblings (SRQ)  (time frame: Change from baseline of the study at 6,12 and 18 months after the randomization; not designated as safety issue).  Satisfaction with the relationship and parenting relationship of the parents (PFB)  (time frame: Change from baseline of the study at 6,12 and 18 months after the randomization; not designated as safety issue).  Eating behaviour of the chronically-ill children/adolescents (EDY-Q)  (time frame: Change from baseline of the study at 6,12 and 18 months after the randomization; not designated as safety issue).  Body-related eating behaviour of the chronically-ill children/adolescents (ChEDE-Q8)  (time frame: Change from baseline of the study at 6,12 and 18 months after the randomization; not designated as safety issue).  Elimination disorders of the chronically-ill children/adolescents (Anamnesebogen Enuresis/Funktionelle Harninkontinenz)  (time frame: Change from baseline of the study at 6,12 and 18 months after the randomization; not designated as safety issue).  Treatment costs of the parents (CSSRI-DE)  (time frame: Change from baseline of the study at 6,12 and 18 months after the randomization; not designated as safety issue).  Treatment costs of the chronically-ill children/adolescents and the siblings (CAMHSRI-DE)  (time frame: Change from baseline of the study at 6 months after the randomization  ; not designated as safety issue).  Treatment assessment (FBB-T)  (time frame: Change from 6 months after randomization at 12 and 18 months; not designated as safety issue).  Patient satisfaction (ZUF-8) Patient satisfaction  (time frame: Change from 6 months after randomization at 12 and 18 months; not designated as safety issue). |
|  | Ethics Review | *The ethics review process information of the trial record in the primary register database. It consists of:*   1. *Status (possible values: Not approved, Approved, Not Available)* 2. *Date of approval* 3. *Name and contact details of Ethics committee(s)* |
|  | Completion date | *Date of excpected study completion:* 31. December, 2022. |
|  | Summary Results | *Not yet availabe, data collection still ongoing* |
|  | IPD Statement | Plan to share IPD: Undecided - It is not yet known if there will be a plan to make IPD available. |
